# Application of Mathematical Modeling and Computational Tools in the Modern Drug Design and Development Process

**DOI:** 10.3390/molecules27134169

**Published:** 2022-06-29

**Authors:** Md Rifat Hasan, Ahad Amer Alsaiari, Burhan Zain Fakhurji, Mohammad Habibur Rahman Molla, Amer H. Asseri, Md Afsar Ahmed Sumon, Moon Nyeo Park, Foysal Ahammad, Bonglee Kim

**Affiliations:** 1 Department of Mathematics, Faculty of Science, King Abdul-Aziz University, Jeddah 21589, Saudi Arabia; rifatmathdu@gmail.com; 2 Department of Applied Mathematics, Faculty of Science, Noakhali Science and Technology University, Noakhali 3814, Bangladesh; 3 College of Applied Medical Science, Clinical Laboratories Science Department, Taif University, Taif 21944, Saudi Arabia; ahadamer@tu.edu.sa; 4 iGene Medical Training and Molecular Research Center, Jeddah 21589, Saudi Arabia; dr.burhan@igene-sa.com; 5 Department of Biological Sciences, Faculty of Science, King Abdul-Aziz University, Jeddah 21589, Saudi Arabia; mrahmanmolla@stu.kau.edu.sa; 6 Biochemistry Department, Faculty of Science, King Abdul-Aziz University, Jeddah 21589, Saudi Arabia; ahasseri@kau.edu.sa; 7 Centre for Artificial Intelligence in Precision Medicines, King Abdul-Aziz University, Jeddah 21589, Saudi Arabia; 8 Department of Marine Biology, Faculty of Marine Sciences, King Abdul-Aziz University, Jeddah 21589, Saudi Arabia; afsar.sumon@gmail.com; 9 College of Korean Medicine, Kyung Hee University, Hoigidong, Dongdaemungu, Seoul 02453, Korea; mnpark@khu.ac.kr

**Keywords:** mathematical modeling, CADD, QSAR, MM-GBSA, MM-PBSA, pharmacophore modeling, MD simulation, biological activity, drug design

## Abstract

The conventional drug discovery approach is an expensive and time-consuming process, but its limitations have been overcome with the help of mathematical modeling and computational drug design approaches. Previously, finding a small molecular candidate as a drug against a disease was very costly and required a long time to screen a compound against a specific target. The development of novel targets and small molecular candidates against different diseases including emerging and reemerging diseases remains a major concern and necessitates the development of novel therapeutic targets as well as drug candidates as early as possible. In this regard, computational and mathematical modeling approaches for drug development are advantageous due to their fastest predictive ability and cost-effectiveness features. Computer-aided drug design (CADD) techniques utilize different computer programs as well as mathematics formulas to comprehend the interaction of a target and drugs. Traditional methods to determine small-molecule candidates as a drug have several limitations, but CADD utilizes novel methods that require little time and accurately predict a compound against a specific disease with minimal cost. Therefore, this review aims to provide a brief insight into the mathematical modeling and computational approaches for identifying a novel target and small molecular candidates for curing a specific disease. The comprehensive review mainly focuses on biological target prediction, structure-based and ligand-based drug design methods, molecular docking, virtual screening, pharmacophore modeling, quantitative structure–activity relationship (QSAR) models, molecular dynamics simulation, and MM-GBSA/MM-PBSA approaches along with valuable database resources and tools for identifying novel targets and therapeutics against a disease. This review will help researchers in a way that may open the road for the development of effective drugs and preventative measures against a disease in the future as early as possible.

## 1. Introduction

A drug is a type of natural or synthetic chemical that is used to prevent, treat, or diagnose disease [1]. It can be able to alter the function of a biological system or target from the molecular to the cellular level. Drug discovery helps to determine new therapeutic candidates by using different computational, experimental, and clinical models. The integrated approaches led to the identification of novel drugs not only from plants but also from other chemical sources [2]. Although various therapeutic compounds originating from plant products are highly regarded, synthetic chemistry and biotechnology products account for the majority of medications in the current medical system [3]. The subject of drug development is exceedingly difficult and needs proper infrastructure and laboratory resources. Unfortunately, the traditional strategy of discovering new drug compounds is a time-consuming process that can take up to 10–15 years and can cost up to USD 2.558 billion to bring a therapeutic to market [4]. This is a multistage and complex process that begins with the identification of an appropriate drug target, followed by drug target validation, hit-to-lead identification, and lead molecule optimization, as well as preclinical and clinical research [5]. Despite the huge financial and time commitments required for medication development, clinical trial success is just 13%, with a high drug attrition rate [6].

A mathematical model is a powerful representation of a biological system that uses mathematical ideas and language to produce an accurate description of the system of principles [7]. The model helps in determining the operation process as well as anticipating certain influencing factors and enables the simulation of complex biological processes that generate hypotheses and suggest experiments [8]. The model also known as forecasting modeling is now frequently used to guide drug development at the industrial level. For example, simulation is the more direct approach that utilizes a mathematical model and predicts system behavior under given conditions [9]. Mathematical model-based biological complex system analysis has high productivity and low cost. The process generates novel lead compounds that undergo clinical trials and reach the market [10]. Most of the major obstacles that arose during the conventional drug design and discovery process may be overcome by employing mathematical models [11]. These models are now being utilized in in silico research to describe various pharmacological properties of potential medicinal drugs [12]. For example, the FDA’s Center for Drug Evaluation and Research (CDER) uses modeling and computer simulations at various phases of drug discovery [13].

Currently, CADD has proven to be a useful and powerful strategy in the manufacture of various medicines [14]. The approach has assisted in overcoming the drawbacks of a time-consuming and expensive procedure in drug research and development [15]. In the latest drug design process, the in silico approach is more important than before. CADD methods such as pharmacophore modeling, virtual screening, molecular docking, and dynamic simulation are frequently applied to identify, develop, and evaluate medicinal properties as well as comparable physiological activity of substances [16]. To quantify the binding efficiency and toxicity of a compound in the classical drug development process, massive in vitro and in vivo trials are required [17]. CADD techniques include a molecular docking methodology that can effectively categorize a large number of molecules with higher binding effectiveness [18]. The method can be used to identify the interaction between a ligand and a receptor at the atomic scale, which helps to identify the binding position of a molecular to a target protein and subsequently provides an idea about the biochemical process [19]. The technique also provides information regarding the target behavior and predicts how a protein (enzyme) interacts with small molecules (ligands) at the binding site of target proteins and facilitates the evaluation of the biological activity of a molecular candidate [20]. Additionally, the CADD approaches include different pharmacology properties analysis tools that can evaluate a compound’s pharmacokinetic (PK) parameters such as bioavailability, toxicity, and effectiveness within a short period. Furthermore, the CADD approaches also include molecular dynamics (MD) simulation techniques that can determine a ligand’s binding stability towards its receptor, which is more suitable and accurate [21]. This review summarizes the challenges associated with target identification in complex biological systems, the benefits of using mathematical approaches, and the ways in which computational models can help consolidate and interpret favorable drug candidates against a specific target as well as disease.

## 2. Target Identification

The early stages of drug discovery probably start with target selection and later move to lead optimization. In the process of potential disease, target discovery is dependent on a variety of resources, involving academic studies, clinical investigations, and the business sector. The pharmaceutical industry, as well as numerous research organizations, use the designated target to locate molecules for developing authorized treatments [22]. Several preliminary stages are involved in this procedure. Throughout the process of target identification and validation, researchers search for chemicals to disrupt a particular biological path that is connected to a certain illness [23]. These compounds can be found in nature, identified through high-throughput screening of large compound libraries, or synthesized as analogs of other drugs that have been proven to be effective against a specific disease. The initial stages in target classification and identification are to determine the function of a possible therapeutic target (which may be a gene or protein) and its involvement in the illness [24]. The molecular processes addressed by the objective are characterized by the following target identification. A good target must always be productive, safe, suitable, and druggable, and it must fulfill clinical and financial requirements. Target identification may be divided into two types: the system biology approach and the molecular biology approach. The system biology approach is a technique that involves studying diseases in complete organisms and selecting targets based on data from clinical trials and in vivo animal research [25]. The molecular biology method, which is at the heart of today’s target identification efforts, aims to find “druggable” targets whose activity may be influenced by associations with molecules, proteins, and sometimes antibodies. Since the biological factors involved in human diseases are so complicated, the foremost essential issue in target identification is not only identifying, optimizing, and choosing trustworthy “druggable” targets, but also truly comprehending the cell membrane associations that identify disease patterns, developing predictive models, and building biological mechanisms for human diseases [26]. For example, G-protein-coupled receptors (GPCRs) and protein kinases are highly “druggable” targets that were identified throughout the molecular biology-based methods [27].

Network-based drug discovery, a field that utilizes information in drug–protein and protein–disease networks, may also be used to study target identification [28]. This strategy entails a highly collaborative scheme between databases and correlations across genomics, transcriptomics, proteomics, metabolomics, the study of the microbiome, and pharmacogenomics, and it is heavily reliant on the development of relevant mathematical, computational, and systems biology tools that connect pharmacological and genomic domains and create computational frameworks for drug target discovery [29]. Another recent network-based application was the combination of large-scale structural genomics and disease association studies to produce a three-dimensional human interactome, which resulted in the identification of candidate genes for previously unknown disease-to-gene associations with proposed molecular mechanisms.

## 3. Mathematical Models in Drug Design

**Figure 1:**
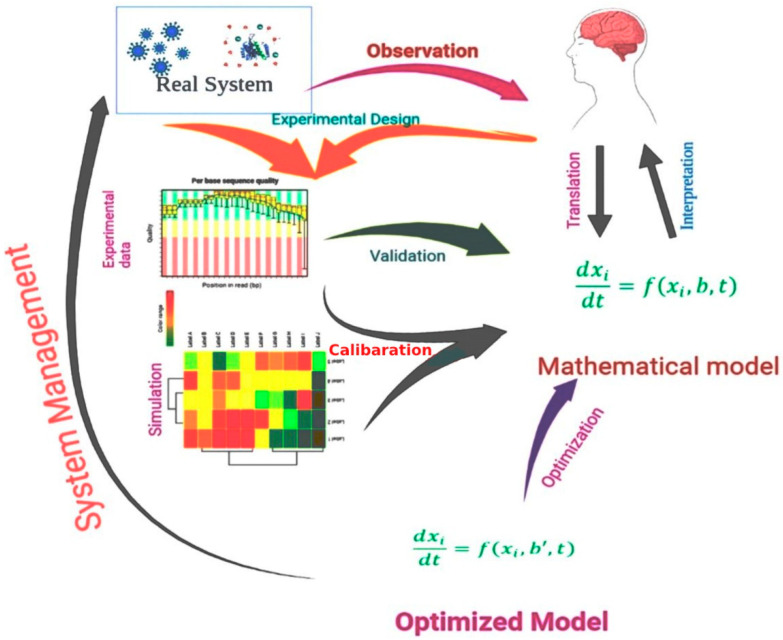
A schematic representation of a mathematical model, including experimental design, experimental data analysis, model optimization, and model validation, used in modern drug design approaches.

Mathematical techniques for drug discovery have a high value because of their potential effect and low cost compared to preclinical studies [30]. The employment of mathematical models, as well as computer simulations, has several advantages. It can be very helpful for systematically determining the relevance of a specific target or pathway for the overall behavior of the system. First, the inconsistencies between the behavior forecasted by a mathematical model and the behavior observed in actual trials might point to missing components, in which the mathematical model allows for a briefer image of a biological mechanism to develop. Although it is not clear which compounds are absent from the system under review, the mathematical model research findings may be used to influence the construction of additional investigations to address the problem. In addition, mathematical models enable a systematic analysis of system fluctuations triggered by the delivery of drugs [31]. However, it is difficult to represent real-world systems such as biological systems in terms of mathematical relationships [32]. Figure 1 shows the process of the use of a mathematical model in the drug design process.

Pharmacokinetic and pharmacodynamic analyses are the earliest and most widely used forms of mathematics in drug design. Pharmacokinetics is the study that describes how drug concentrations change over time, whereas pharmacodynamics explains how drug effects fluctuate with concentration. Pharmacokinetics depict a possible drug’s concentration in the appropriate organ compartments (e.g., circulating blood). Pharmacodynamic models relate this concentration to a biomarker that is thought to be linked with a disease state, often considering the modification of the pharmaceutical target [33].

Cancer research is a good example of how mathematical models are used in drug discovery. One of the most widely employed mathematical models in cancer treatment research is integrated into network-based medicine [34]. Network medicine is a discipline of medicine that explores molecular and physiological links with therapeutic implications. Infectious diseases, such as malaria, are another instance of a mathematical model application in drug innovation [35]. In this situation, mathematical models may be employed to evaluate the prospective drug’s capacity to destroy the parasite at a different phase of the disease. Compound pharmacokinetics and compound pharmacodynamics are used in such models. COVID-19, an infectious viral disease, is the most recent example of how mathematical models are employed in drug discovery [36].

## 4. Target Validation

Target validation demonstrates that a cellular target is actively engaged in a disease state and that modulating the target is likely to be therapeutic. The use of a multiple-validation strategy is the most significant criterion for target validation. Pathways are included in target validation. Drug discovery benefits from research targeted at identifying targets that govern and regulate activities in living organisms [37]. An examination of target validity, or if the molecule targets a biological component relevant to the disease, will be critical to moving any molecule forward. Is the target expressed in the human brain during the disease process, providing for a therapeutic window? Researchers can better understand side effect profiles by understanding routes and their relationships. Because most diseases are the consequence of interactions between several stages and elements, any modification made at a different stage or in various tissues will result in different outcomes [38]. As a result, the associated treatments should differ as well. As a result, identifying the route can show some slight changes between diseases that have similar symptoms. Finding these distinctions is critical in the drug development process since it allows scientists to create different compounds for different diseases [39].

## 5. Mathematical and Computational Biology Approaches for Target Validation

Before moving on to the preclinical phases of drug development, mathematical biology models may create a new desired location and look to comprehensively evaluate the goal early in the investigation [40]. This target space can be wider than what is now considered a prospective target by using a less reductionist approach. Target validation will be a continual process if mathematical biology techniques are used from the beginning [41]. Preclinical target verification will be viewed as a bundle that comprises the following factors: A suitable target might be demonstrated to alter illness and play a significant role in the pathological process early in the phase of drug development. It includes a mechanism investigation of the target’s activity in a system, as well as an early investigation of the target’s modifications and consequences in clinical samples.

## 6. Protein Structure Prediction

**Figure 2:**
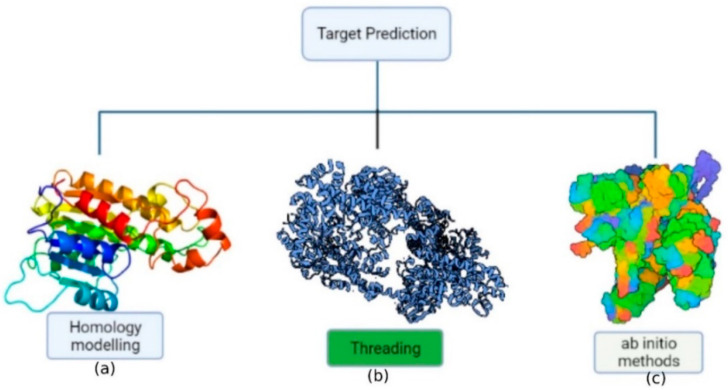
Representation of the protein structure prediction methods: (**a**) homology-based approach; (**b**) threading approach; (**c**) ab initio approach.

Proteins are vital molecules that are involved in a variety of biological activities. Protein structure prediction or modeling is critical since a protein’s activity is largely determined by its three-dimensional structure. Furthermore, a protein’s 3D structure is determined by its amino acid composition. Experiments using X-ray crystallography or NMR spectroscopy to resolve protein structure are time-consuming, expensive, and complex [42]. Consequently, theoretical knowledge of protein structure, dynamics, and folding has been used to construct a model from amino acid sequences due to the improvement of computer methods and computational tools. The approaches for predicting protein structure may be divided into three categories (Figure 2): (a) homology modeling; (b) threading; (c) ab initio methods (de novo).

The most effective computer technique for protein structure prediction is homology modeling, which involves predicting an unknown structure using a similar known protein structure as a framework [43]. An ideal therapeutic simulation of a protein may be built by assigning a structure based on sequence alignment and then creating the model and minimizing energy. Despite homology modeling’s predictive potential and utility, some issues remain. Firstly, the amounts of target-template architectural conservation and alignment precision are key indicators of the model’s quality. If the identity of the template sequence is below 20%, around 50% of residues inside the layout are likely to be misaligned. Another concern includes that homology modeling systems should develop innovative ways to manage the expanding number of existing protein molecules. To date, different homology modeling tools has been developed and the most frequent use tools use for the modeling has been listed in Table 1.

Threading a sequence throughout a fold involves a precise adjustment of the protein’s amino acid sequence with the folding motif’s corresponding amino acid residue residues. The main goal of this technique is to determine the most possible fold from a given sequence or to find appropriate sequences that might fold into a certain structure. Threading performance is characterized by the number of useable folds whose structures are determined precisely towards the atomic level [44]. Threading processes, which use approaches for aligning sequences with 3D shapes to determine the proper folding of a given sequence from a range of possibilities, were used to make the predictions.

In absence of an experimentally solved structure of a similar/homologous protein, ab initio (de novo) protein structure prediction is a technique for evaluating the three-dimensional structure, when an experimentally solved structure of a similar/homologous protein is not present. The energy function guides the construction of protein structure in this strategy. The ab initio (from scratch) methodologies are based on first-principles physics and chemistry regulations, as well as the premise that a protein’s natural structure always remains at the lowest energy level [45]. However, the precision of ab initio modeling is poor, and performance is generally limited to tiny proteins (120 residues).

**Table 1:** Summary of the most widely recognized homology modeling tools use in drug development.

No	Name	Application	Availability	Reference
1.	I-TASSER	Reassembling fragment structure via threading	https://zhanggroup.org/I-TASSER/	[46]
2.	SWISS-MODEL	Segment assembly/local similarity	https://swissmodel.expasy.org/	[47]
3.	ESyPred3D	3D modeling, template identification, and alignment	https://www.unamur.be/sciences/biologie/urbm/bioinfo/esypred/	[48]
4.	HH-suite	Template detection, alignment, 3D modeling	https://arquivo.pt/wayback/20160514083149/http:/toolkit.tuebingen.mpg.de/hhpred	[49]
5.	RaptorX	Protein 3D modeling, remote homology discovery, and binding site prediction	http://raptorx.uchicago.edu/	[50]
6.	FoldX	Protein design and energy calculations	https://foldxsuite.crg.eu/	[51]
7.	ROBETTA	Rosetta homology modeling and fragment assembly from scratch with Ginzu domain prediction	http://robetta.bakerlab.org/	[52]
8.	BHAGEERATH-H	Methods of ab initio folding and homology are combined	http://www.scfbio-iitd.res.in/bhageerath/bhageerath_h.jsp	[53]
9.	Prime	Homology modeling, evaluation, and refining of the produced model using the energy function	https://www.schrodinger.com/prime	[54]
10.	LOMETS	Tertiary structure prediction with a local meta-threading server	https://zhanglab.ccmb.med.umich.edu/	[55]

## 7. Computer-Aided Drug Design

**Figure 3:**
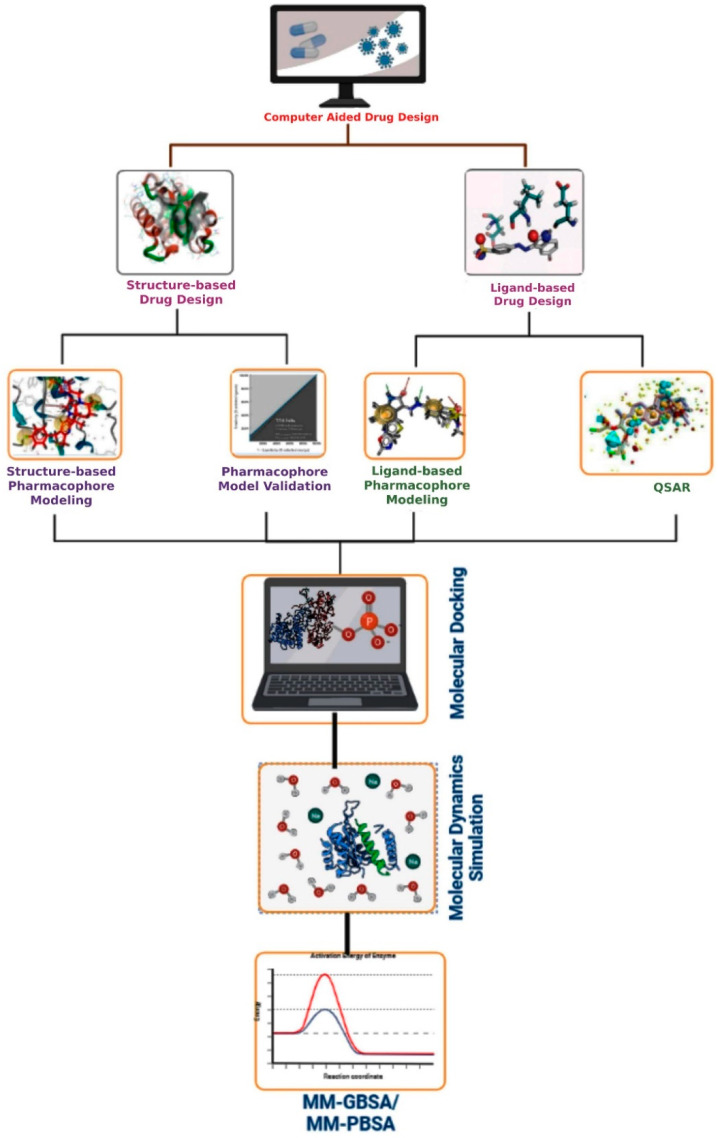
Representation of the basic workflow of computational drug design approaches. The CADD approaches include structure- and ligand-based drug design approaches, pharmacophore modeling, virtual screening, molecular docking, ADMET, dynamics simulation, and MM-GBSA or MM-PBSA approaches.

Computer-aided drug design methods have been applied in the field of drug development over the past two decades [56]. Currently, this is seen as one of the best appropriate alternatives to high-throughput screening, which is routinely used in drug design and development. CADD may be used for all efforts that have been made throughout the process of drug development that can be described mathematically and analyzed using numerical methods [57]. Figure 3 demonstrates the basic CADD approach that may be utilized interactively with experimental methodologies to find novel drug targets and direct iterative ligand optimization. Structure-based and ligand-based drug design techniques are two types of CADD that have been widely used throughout the development of drugs process to find acceptable lead compounds. The CADD approaches help to expedite the drug discovery and development process by minimizing the cost and time [58]. However, if the computer system crashes unexpectedly, the CADD designs might be lost. If proper precautions are not performed, viruses will infect the computer system.

### 7.1. Structure-Based Drug Design

Structure-based drug design (SBDD) (or direct techniques) can be used if the target’s spatial structure is available. Compounds with qualities complementary to the target area can be created based on the properties and features of the macromolecule’s spatial structure. X-ray crystallography, NMR, and in silico homology-based prediction approaches can all be used to determine a protein’s 3D structure. The protein’s binding/active site is discovered when the three-dimensional structure is understood. Structure-based pharmacophore modeling, virtual screening (SBVS), molecular docking, and molecular dynamics (MD) simulations are some of the typical methodologies used in SBDD.

#### 7.1.1. Structure-Based Pharmacophore Modeling

The pharmacophore features are discovered by utilizing the shape of the complicated molecular target [59]. The characteristics are founded on a single X-ray crystallized target–ligand complex. The pharmacophore characteristics are built using a single ligand as well as its associations with the specific target protein. The fundamental contrast between ligand-based and structure-based approaches is the number of ligands utilized to construct the pharmacophore. The ligand-based technique necessitates at least 30 actives, whereas the structure-based method necessitates only one ligand and its connection with the receptor. Furthermore, the pharmacophore technique is derived from an active site of the ligand. Another method for creating a structure-based pharmacophore is to employ an APO template in such a way that the active site amino acids are determined and then develop a feature list based on their interaction properties that may be included in the pharmacophore [60]. The only drawback is when the list predicts too many features (more than seven features).

#### 7.1.2. Pharmacophore Model Validation

Structure-based pharmacophore modeling can be employed efficiently when there is inadequate information on ligands that have been empirically proven to inhibit or stimulate the activity of a certain therapeutic target [61]. Validation is required to obtain an accurate pharmacophore analysis and to analyze the molecular model’s quality. Pharmacophore methods focused on appropriate correlation coefficients (R) might be validated in three main steps: Fisher’s randomization test, test set prediction, and Guner–Henry (GH) scoring technique.

##### Fisher’s Randomization Test

Fisher’s randomization approach is critical for establishing a link between structural and biological functionality in training set molecules [62]. The relevant experimental data linked with the training dataset are randomly changed to make them statistically irrelevant. The randomized dataset is then used to construct assumptions using the same characteristics and variables that were used to develop the original hypothesis. This randomization approach validated the drug-tested pharmacophore hypothesis by selecting 95% confidence levels, which resulted in 19 random spreadsheets. The randomized dataset should give equivalent or higher cost values, improved RMSD, and significant correlations for successful pharmacophore development.

##### Test Set Prediction

The goal of the pharmacophore method is to anticipate not only the behavior of molecules in the training dataset, but also the activity of external molecules. The correlation value between the experimental and forecasted behavior of external molecules that were excluded from the training dataset was predicted using test set prediction. This metric determines the predictability of pharmacophores’ stability (free of errors). In this technique, the behavior of the test set components has a higher correlation coefficient, which has a 95% confidence level [63].

##### Guner–Henry (GH) Scoring

The basic goal for implementing a decoy set is the validation of a pharmacophore model to see how effectively it can distinguish the active and inactive compounds. The validation of the model depends on a scoring function known as the Guner–Henry (GH) score. The GH score ranges from 0 to 1, where 1 indicates the most optimum model. The GH score can be calculated based on the following formulas [64]:

(1) $$\%A=\frac{\mathrm{Ha}}{A}\times 100$$

(2) $$\%Y=\frac{\mathrm{Ha}}{\mathrm{Ht}}\times 100$$

(3) $$\mathrm{EF}=\frac{\mathrm{Ha}/\mathrm{Ht}}{A/D}$$

(4) $$\mathrm{GH}=\left(\frac{\mathrm{Ha}\left(3A+\mathrm{Ht}\right)}{4\mathrm{HtA}}\right)\left(1-\frac{\mathrm{Ht}-\mathrm{Ha}}{D-A}\right)$$

where, D is the number of the compound, A is the number of the active compound, Ht is the number of hits retrieved, Ha is the active hit, %A is the ratio of actives retrieved, %Y is the hit relative fraction to the size of the database (hit rate or selectivity), EF is the enrichment factor, and GH is Guner–Henry score.

#### 7.1.3. Virtual Screening

The structure-based virtual screening (SBVS) strategy is the most extensively utilized strategy in in silico drug discovery [65]. SBVS employs evaluating functions to measure the force for non-covalent contacts between ligand and biological target, and it tries to predict the optimum collaboration between two compounds to create a sustainable complex [66]. The benefit of SBVS is that it not only reduces the time but also reduces the expense of screening millions of small compounds [67]. Because the molecule’s physical presence is not required, it may be computationally evaluated before being produced. On the other hand, because of the challenges in calculating the complexity of ligand–receptor binding interactions, it is hard to precisely anticipate the right binding location, which is one of SBVS’s shortcomings. Sometimes, it has the potential to produce not only false positives but also false negatives at the same time. In addition, it has been demonstrated that the presence of stereochemical and valence errors in the chemical data libraries could also cause investigators to choose unfeasible compounds

#### 7.1.4. Molecular Docking

The molecular docking approach can be used to illustrate the atomic-level interplay between small molecules and proteins in an attempt to characterize small-molecule behavior at target protein binding sites and describe basic biochemical mechanisms [68]. Shapes, electrostatic interactions, hydrogen bonds, and van der Waals and Coulombic interactions are all taken into consideration during docking [69]. Molecular docking research is possible between protein and protein, protein and ligand, and protein and nucleotide that can be performed by using any of the tools listed in Table 2 [70]. A docking score indicates binding potentiality, and various methods of fitting the ligand into the binding site are investigated [71]. Flexible-ligand search docking and flexible-protein docking are the two major forms of molecular docking [72]. In the case of flexible-ligand search docking, three techniques are generally used, namely the systematic approach, the stochastic method, and the simulation method, while flexible-protein docking typically uses Monte Carlo (MC) and molecular dynamics (MD) methods [73]. The methods have many advantages in CADD approaches, but the lack of confidence in the ability of scoring functions to give accurate binding energies is one of the major limitations of molecular docking.

### 7.2. Ligand-Based Drug Design

Ligand-based drug design is considered an indirect technique because the structure of the biomolecular target is unknown and cannot be anticipated using approaches such as homology modeling [87]. The most significant and highly used methods in ligand-based drug discovery are 3D quantitative structure–activity relationships (3D QSARs) and pharmacophore modeling, both of which can supply vital knowledge regarding the nature of connections between drug targets and ligand compounds as well as computer simulations suitable for lead compound optimization [88]. The most crucial aspects of the interaction nature are preserved, but the noise of extra information is eliminated.

#### 7.2.1. Quantitative Structure–Activity Relationship (QSAR) Models

Structure–activity analysis relationship models depict the overall mathematical relationship between a collection of chemicals’ structural properties and target response [89]. The QSAR model has been successfully employed to decrease the need for time-consuming, arduous, and expensive processes in innovative drug development during the last few decades, and it also performed well in terms of predicting physiochemical properties (Table 3). Regression techniques, artificial neural networks, principal component analysis (PCA), and partial least squares (PLS) can be used to determine these correlations. Multiple linear regression is a frequently used approach for establishing a link between active and multiple structural features. When a high number of structural features must be taken into account (for example, grid-based approaches in 3D QSAR), linear regression fails and a specialized method such as PCA or PLS is needed. The idea of multidimensional QSAR has been proposed in recent years [90]. Predicting the biological properties of chemical substances is more beneficial. HQSAR, G-QSAR, MIA-QSAR, and multitarget QSAR are all part of this process, which has had outstanding success in the new drug process. The two most essential methodologies suggested for developing pharmacological compounds are comparative molecular field analysis (CoMFA) and comparative molecular similarity indices analysis (CoMSIA). However, QSAR modeling has some limitations; for example, if the number of molecules in the training set is small, the data may not accurately reflect all of the properties, and therefore it cannot be used to forecast the most active compounds.

#### 7.2.2. Ligand-Based Pharmacophore Modeling

Pharmacophore mapping is a crucial part of ligand-based drug discovery and development [98]. Medication must have functional groups that are arranged in a precise way to generate a particular biochemical response. The pharmacophore is defined by this pattern throughout the drug design process. The active molecules in a ligand-based pharmacophore are loaded in such a manner that their biochemical properties are imposed as much as feasible. Molecule alignment may be accomplished differently, using rigid approaches that need knowledge of ligand active conformations not only from the semi-flexible method but also from flexible methods [99]. The pharmacophore model is made up of a set of chemical properties (such as H-bond acceptors and donors; charged or ionizable groups; hydrophobic or aromatic rings; and physical features in terms of length, angles, and dihedrals) that are shared by a group of mixtures with strong inhibitory mechanisms and required for their inhibition effect against a specific objective [100]. Most popular pharmacophore modeling tools invented to date has been represented in Table 4.

## 8. Pharmacokinetics Property Analysis

**Figure 4:**
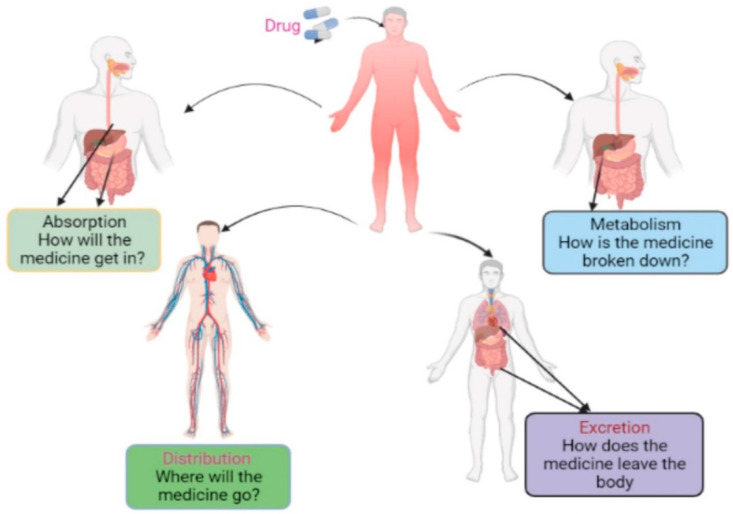
Schematic representation of ADME approaches required during the drug design process. Herein, the absorption, distribution, metabolism, and excretion process utilized by a drug in human systems are explained.

The term “pharmacokinetics” originated from the Greek words pharmakon (drug) and kinetikos (movement) and describes the investigation of the dynamic movements of foreign chemicals (xenobiotics) throughout the body, including absorption, distribution, biotransformation/metabolism, and excretion (ADME), as shown in Figure 4. It may simply be defined as the body’s response to xenobiotics that can be evaluated by using the tools listed in Table 5 [111]. The distribution of therapeutic drugs in an organism is characterized by ADME, a well-known and recognized pharmacology concept. The word “pharmacokinetic” refers to the ADME/T between a pharmacological substance in pharmacology. More than half of all medication candidates fail preclinical trial tests due to insufficient ADME characteristics [112]. Recent methodologies and improvements in the drug detection process have led to a large number of potential therapeutic compounds that are currently undergoing preclinical ADMET evaluation.

### 8.1. Absorption

The transfer of medicine from its delivery site into the circulation is referred to as absorption. Absorption describes how much drugs are absorbed and how much time is necessary for absorption; the quantity entering the bloodstream in an unmodified state is known as bioavailability [113]. Multiple variables influence the rate and scope of medication absorption, including administration route, a drug’s formulation and chemical qualities, and food–drug interactions. There are two fundamental routes for drug absorption once the medication is present in solution form: active transport and passive diffusion. Chemical carriers in the membrane bind to drug molecules and transport them through the membrane to the opposite side, where they are discharged [114]. Because the membrane plays an active part in this process, it is called active transport. Chemical energy is required, and molecules can be moved from a low-concentration zone to a higher-concentration region. During passive diffusion, the membrane plays a passive role in drug absorption; most medicines pass through the membrane this way. The physicochemical properties of the drug as well as the intensity of the drug gradient across the membrane impact the rate of drug transfer.

### 8.2. Distribution

The technique of drug distribution is important because it impacts how much medication enters the active areas, and it measures drug efficacy and toxicity. Drug distribution is mostly determined by the binding and unbound forms of enzymes and proteins found in the circulation. The binding of medication with plasma proteins influences its effective distribution [115]. If less medication binds with plasma proteins, more drugs are distributed across cell membranes, resulting in better bioavailability. A medication will travel from the site of absorption to tissues throughout the body, including the brain, fat, and muscle. Tissues and organs with a large blood supply, such as the heart, liver, and lungs, are one such location. The center volume of distribution indicates this space. The opposite part has a lower blood supply. The sum of tissue spaces not included in the core volume is referred to as the peripheral volume of distribution. The center volume is distributed first, followed by the peripheral volume. Smaller molecules readily pass through biological membranes and obtain a much greater percentage of distribution, while bigger molecules struggle to penetrate the cellular membrane and yet have a lower probability of distribution [116].

### 8.3. Metabolism

Once a medication enters an organism’s body, the activity of catabolism and anabolism defined as metabolism begins with the assistance of different enzymes accompanied by different chemical components and solvent processes [117]. The primary goal of drug metabolism is simply to convert these drug molecules into further polar, water-soluble stages or end products that can be easily eliminated from an organism’s body. Drugs can indeed be metabolized by the processes of oxidation, reduction, hydration, conjugation, concentration, or isomerization, making the drug easier to eliminate. Although enzymes are found in many tissues that are engaged in the metabolism process, the liver contains the largest quantity [118]. Drug metabolism processes vary amongst individuals. Some people metabolize drugs so fast that bioactive bloodstream concentrations are not achieved; others’ metabolism is extremely slow, rendering usual dosages toxic.

**Table 5:** Summary of the most usually recognized ADME analysis tools used in the computational drug design process.

S. No.	Program	Description	Accessibility	Reference
1.	ADMETlab	ADMET in a systematic manner utilizing the ADMET database	http://admet.scbdd.com/	[119]
2.	eMolTox	Molecular toxicity prediction	http://xundrug.cn/moltox	[120]
3.	LIVERTOX	Hepatotoxicity prediction	https://livertox.nih.gov/	[121]
4.	vNN	ADMET forecasts	https://vnnadmet.bhsai.org	[122]
5.	PreADMET	This online tool calculates the probability of carcinogenicity as well as poisonous potency	https://preadmet.bmdrc.kr/	[123]
6.	QikProp	Used to forecast ADMET-related features	https://www.schrodinger.com/qikprop	[124]
7.	SwissADME	Estimate physicochemical characteristics and predict ADME	http://www.swissadme.ch/	[125]
8.	DSSTox	It is a public database of searchable distributed structure toxicity	https://comptox.epa.gov/	[126]
9.	ChemTree	It is used to forecast ADMETox characteristics.	https://chemtree.kr/	[127]
10.	Metabase	It is a low-cost radio analytical LIMs in ADME/PK research based on Excel	https://www.metabase.com/	[128]
11.	TOPKAT	Used in toxicology prediction	https://www.toxit.it/en/services/software/topkat	[129]

### 8.4. Excretion

Excretion is the process of removing or eliminating undesirable products or molecules from an organ system. The liver and kidneys are the principal sites for drug excretion; however, the skin, lungs, bile, and stomach may also be involved. The components, whether metabolized or unmetabolized, might be eliminated from the individual’s system. The total complicated elimination process is performed by the kidneys involving urine and occasionally sweat.

## 9. Toxicity Analysis

Toxicity evaluation is an important process before a drug candidate goes through a clinical trial for improved lead chemical selection [130]. Toxicity is a measurement of any undesirable or unfavorable molecules or substances that impact the human body or system. To investigate the toxicity of substances, the traditional drug design process includes several animal studies, which are time-consuming, expensive, and require ethical concerns. Computer-aided toxicity tests, as compared to traditional approaches, are quick and economical ways to remove potentially harmful chemical compounds and minimize the number of biological experimental procedures [131]. Genotoxicity, carcinogenicity, skin sensitization, irritation, ecotoxicity, and other endpoints are used to calculate toxicity [132]. Single or multiple dosage studies are used to determine the effects of chemicals on humans, animals, plants, or the environment. Blockage of human ether-à-go-go-related gene (hERG) potassium ion channels, for example, is toxic to the heart and can cause serious cardiac arrhythmia. As a result, early identification of suspected hERG inhibitors or non-inhibitors may play a critical role in decreasing cardiotoxicity.

## 10. Molecular Dynamics Simulation

Molecular dynamics (MD) simulation itself is a computer-based simulation approach that focuses on analyzing the movement of atoms and molecules through a conformational space. The stability of protein–ligand complexes in a certain artificial environment may be confirmed via MD modeling. As a result, researchers ran a 50 ns MD simulation to examine the protein–ligand complexes’ steady-state nature and conformational stability [133]. MD uses Newton’s second rule of motion to anticipate novel conformations of a molecular system by integrating over time the force exerted on the system and the velocities of the atoms in the system. MD simulation techniques minimize the probability of a molecular system becoming stuck in a local lowest energy area during a simulation, allowing for complete conformational space sampling [134]. Consequently, molecular dynamics simulation is still a better, quicker, more rational, and more broadly accessible process for drug design using in silico methods [135]. Additionally, the position and velocity of each atom in the system are caught at every moment in time, which is difficult to do with any experimental approach. It generally describes the protein’s atomic and molecular characteristics, drug–target interactions, chemical solvation, and conformational adjustments that a receptor exhibits under different situations. However, the main limitation is in simulated time, which at present is in the order of nanoseconds for a large system.

## 11. MM-GBSA/MM-PBSA

Molecular mechanics Poisson–Boltzmann surface area (MM/PBSA) and molecular mechanics generalized Born surface area (MM/GBSA) are the most renowned methods for MD snapshots that can calculate a single minimized structure from many structures. One of the significant advantages is the ability to split complete available energy via sub-components and separately quantify individual contributions using MM/PB(GB)SA. This feature is unquestionably beneficial when evaluating various free-energy approaches. MM/PB(GB)SA may be used in a variety of configurations, including microscopic host machines to giant protein–protein interactions involving hundreds of molecules [136]. The whole approach is mostly employed in docking studies that require a rapid assessment of binding affinities. Docking software and servers use a scoring system that takes binding affinity into account when determining the feasible configurations of such ligand inside a binding pocket. MM/PB(GB)SA may enhance the effectiveness of such scoring methods [137]. MM/GBSA, for example, was used to increase the effectiveness of molecular docking software in the Drug Design Data Resource and Grand Challenge 4. A huge dataset of protein–ligand complexes with non-redundant binding poses was used to predict proper configurations using MM/GBSA [138]. Recently, MM/GBSA was used to investigate the effect of nelfinavir stereoisomers on the SARS-CoV-2 main protease [139].

## 12. Conclusions

Identification and characterization of drug targets and their corresponding active compounds are largely dependent on mathematical and computational methodologies and complicated systems biology tools. By using these approaches, chemical and structural characterization of the molecules is made possible, thus leading to a reduction in the risk of complications and failure of drug candidates. Traditional experimental studies for designing and developing drugs are expensive and time-consuming processes that can be optimized by using different mathematical models and computational tools. Therefore, the use of computational tools and mathematical modeling is increasing day by day for molecular modeling and therapeutics discovery. However, more mathematical and computational study is necessary to eliminate individual bias in the design and development of lead compounds. Furthermore, these methodologies have a high impact on the system biology and drug design and development process, which can help to identify more accurate targets as well as target specific drugs. However, more emphasis should be focused on the development of new and more accurate tools that can be used to refine the existing molecular approaches for future drug design processes.

## Figures and Tables

**Table 2:** Summary of the most widely recognized molecular docking software used across the computational drug design process.

No.	Programs	Application	Accessibility	Reference
1.	AutoDock	It is employed in molecular docking. It predicts the binding capacity of a tiny chemical and assigns a target protein to a 3D structure	https://autodock.scripps.edu/	[74]
2.	LPCCSU	Based on a comprehensive investigation of interatomic interactions and interface complementarity	https://oca.weizmann.ac.il/oca-bin/lpccsu	[75]
3.	PatchDock	The method performs rigid docking, with surface variability	https://bioinfo3d.cs.tau.ac.il/PatchDock/php.php	[76]
4.	Hex	For docking studies	http://hex.loria.fr/	[77]
5.	Glide Schrodinger	Comprehensive molecular modeling and computer-aided drug development (CADD) tool	https://www.schrodinger.com/	[78]
6.	Molecular operating environment	Comprehensive molecular modeling and computer-aided drug development (CADD) tool	https://www.chemcomp.com/	[79]
7.	DockingServer	A user-friendly web-based interface that manages all elements of molecular docking.	https://www.dockingserver.com/web	[80]
8.	SwissDock	A web service for predicting a protein’s association with a small molecule ligand.	http://www.swissdock.ch/	[81]
9.	LeDock	A molecular docking program for docking ligands with protein targets	http://www.lephar.com/software.htm	[82]
10.	MedusaDock 2.0	Fast flexible docking with a discrete rotamer library of ligands	https://dokhlab.med.psu.edu/cpi/#/MedusaDock	[83]
11.	Molegro Virtual Docker	Based on a novel heuristic search method that integrates differential evolution and a cavity prediction algorithm	http://molexus.io/molegro-virtual-docker/	[84]
12.	MOLS 2.0	Using mutually orthogonal Latin squares, induced-fit peptide–protein, and small molecule–protein docking	https://sourceforge.net/projects/mols2-0/	[85]
13.	ParaDockS	Metaheuristics for population-based molecular docking	http://www.paradocks.at/	[86]

**Table 3:** A list of techniques and mathematical equations used in QSAR modeling as well as drug design.

No	Techniques	Equation	Activity	Reference
1.	K-nearest neighbor	Linear	Simple	[91]
2.	Multiple linear regression	Linear	Simple	[92]
3.	Partial least squares	Linear	Performs effectively on data including a big dataset	[93]
4.	Artificial neural network	Nonlinear	Works well with nonlinear data	[94]
5.	Support vector machine	Nonlinear	A most effective approach for classification and regression	[95]
6.	Decision tree	Nonlinear	Extremely interpretable	[96]
7.	Random forest	Nonlinear	A better and more reliable estimate	[97]

**Table 4:** Summary of the most usually recognized pharmacophore modeling software used in drug development.

No.	Programs	Application	Accessibility	Reference
1.	Align-it	Pharmacophore alignment	http://silicos-it.be/	[101]
2.	Catalyst	Pharmacophore modeling	http://accelrys.com/products/discovery-studio/pharmacophore.html	[102]
3.	MOE	Pharmacophore modeling	http://www.chemcomp.com/MOE-Pharmacophore_Discovery.htm	[103]
4.	LigandScout	Pharmacophore modeling	http://www.inteligand.com/ligandscout/	[104]
5.	Phase	Pharmacophore modeling	http://www.schrodinger.com/Phase/	[105]
6.	Quasi	Pharmacophore modeling	http://www.denovopharma.com/page2.asp?PageID=485	[106]
7.	Pharmer	Pharmacophore search	http://smoothdock.ccbb.pitt.edu/pharmer/	[107]
8.	Open3DQSAR	Exploration of pharmacophores using high-throughput chemometric analysis	http://open3dqsar.sourceforge.net/	[108]
9.	Pharmagist	A website for the discovery of ligand-based pharmacophores	https://bioinfo3d.cs.tau.ac.il/PharmaGist/	[109]
10.	FLAP	The fingerprints are characterized by pharmacophoric properties	https://www.moldiscovery.com/software/flap/	[110]

## Data Availability

Not applicable.
